# Die ersten Schritte nach einer hüftnahen Fraktur: Sensorbasierte Bewegungsexploration bei geriatrischen Traumapatient/-innen

**DOI:** 10.1007/s00391-021-01861-3

**Published:** 2021-02-23

**Authors:** Amelie Altenbuchner, Sonja Haug, Karsten Weber

**Affiliations:** 1 Institut für Sozialforschung und Technikfolgenabschätzung (IST), Ostbayerische Technische Hochschule Regensburg (OTH), Seybothstraße 2, 93053 Regensburg, Deutschland; 2 Fakultät Angewandte Sozial- und Gesundheitswissenschaft, Institut für Sozialforschung und Technikfolgenabschätzung (IST), Ostbayerische Technische Hochschule Regensburg (OTH), Regensburg, Deutschland; 3 Institut für Sozialforschung und Technikfolgenabschätzung (IST), Regensburg Center of Health Sciences and Technology (RCHST), Ostbayerische Technische Hochschule Regensburg (OTH), Regensburg, Deutschland

**Keywords:** Schrittzahlen, Rehabilitation, Mobilität, Motion-Tracker, Alterstraumatologie, Step numbers, Rehabilitation, Mobility, Motion tracker, Geriatric traumatology

## Abstract

**Hintergrund:**

Sensoren erlauben eine durchgehende Mobilitätsbeobachtung von Patienten nach hüftnahen Frakturen. Fitnessarmbänder ermöglichen unterbrechungs- und einschränkungsarme Längsschnittbeobachtungen der geriatrischen Zielgruppe.

**Ziel der Arbeit:**

Deskriptive Darstellung der Schritte pro Tag (SpT) nach Hüftfrakturoperation auf der geriatrischen Traumastation und darüber hinaus.

**Material und Methoden:**

Die explorative Studie mit Feldcharakter beobachtete über 10 Wochen die SpT von 20 Patienten (80 % weiblich, Durchschnittsalter 85,2 Jahre ± 7,86) via Fitnesstracker. Tägliche Schrittzahlen (SpT) werden für Patienten als wöchentlicher Durchschnittswert (Tage 1–7, 8–14 usw.) gebildet, bevor die deskriptive Analyse erfolgt.

**Ergebnisse:**

Es erfolgt eine positive Entwicklung im Laufe der Beobachtungswochen (BW). Von BW 1 (*M* = 353,57 ± 310,15) bis 10 (*M* = 2482,07 ± 1374,12) erfolgt ein durchschnittlicher Anstieg um den Faktor 1,285 (±0,351). Die höchsten Anstiege um das je 1,8-Fache sind von BW 2 (*M* = 556,27 ± 478,11) auf BW 3 (*M* = 1024,86 ± 921,24) sowie von 6 (*M* = 1268,21 ± 880,47) auf 7 (*M* = 2367,14 ± 1680,08) zu beobachten. Geringfügig verringern sich die Schritte von BW 4 (*M* = 1208,27 ± 1210,45) auf 5 (0,99-fach) und von BW 9 (*M* = 2689,98 ± 2339,71) auf 10 (0,92-fach). Es sind stets hohe Spannweiten sowie Standardabweichungen in Anbetracht der Mittelwerte vorhanden. Es lässt auf das Vorhandensein mehrerer Gruppen der Schritteentwicklung schließen.

**Diskussion:**

Fitnesstracker und die Variable SpT können die Gehfähigkeit in der Alltagsumgebung abbilden, bei einem möglichen Unterschätzen < 10 %. Es treten Unterschiede in Beobachtungsdauer sowie -unterbrechungen auf. Eine Clusteranalyse sollte zukünftig Gruppenmerkmale unterschiedlicher Entwicklungsverläufe aufdecken.

## Abstract (en)

**Background:**

Sensor-based monitoring allows continuous observations of patient mobilization after proximal femoral fractures. A wrist-worn motion tracker allows long-term observation that is low in interruption and constraints for subjects.

**Objective:**

Description of steps development after hip fracture surgery on a specialized geriatric trauma ward and beyond.

**Material and methods:**

In the explorative long-term field research study, an applicable motion tracker observed steps per day of 20 patients (80% female, mean age 85.2 years ± 7.86 years) for 10 weeks. Weekly mean values (days 1–7, 8–14 etc.) of steps per day formed the database for descriptive analysis (mean, SD, min, max, median).

**Results:**

During observation weeks (ow) a positive development of steps took place. A mean increase factor of 1.285 (±0.351) occurred from ow 1 (*M* = 353.57 ± 310.15) to ow 10 (*M* = 2482.07 ± 1374.12). The highest increase by a factor of 1.8 could be reported from ow 2 (*M* = 556.27 ± 478.11) to ow 3 (*M* = 1024.86 ± 921.24) as well as from ow 6 (*M* = 1268.21 ± 880.47) to 7 (*M* = 2367.14 ± 1680.08). A slight decrease of steps occurred from ow 4 (*M* = 1208.27 ± 1210.45) to ow 5 (0.99-fold) and from ow 9 (*M* = 2689.98 ± 2339.71) to 10 (0.92-fold). High ranges and standard deviations in relation to the mean occurred constantly. The presence of several step development groups could be presumed.

**Conclusion:**

Motion tracker and the variable steps per day can represent the ability to walk within an everyday environment, with a possible underestimation of < 10%. Differences regarding observation lengths and disruptions occurred. Cluster analysis should detect group attributes of different courses of development in subsequent studies.

Nach einer Fraktur ist Mobilisierung Behandlungsziel und Therapiesäule. Das Festlegen von Outcomes basiert jedoch auf vielen Unsicherheiten, da Assessments nicht für alle Patient/-innen geeignet sind. Sie können tagesabhängig beeinflusst und subjektiv geprägt sein. Sensorbasiertes Bewegungsmonitoring bietet eine Ergänzung zur Operationalisierung der Gehfähigkeit. Für Längsschnittuntersuchungen, die auch im häuslichen Umfeld durchgeführt werden, eignet sich die tägliche Schrittzahl als Variable. Sie kann durch einen handelsüblichen Fitnesstracker beobachtet werden.

## Hintergrund und Fragestellung

Mobilität als Ausdruck der körperlichen Fähigkeit, sich fortzubewegen – also mit oder ohne Hilfsmittel zu gehen –, ist für geriatrische Patient/-innen essenziell. Sie beeinflusst den Gesundheitsgrad und die Lebensqualität positiv und wirkt präventiv gegen eine Erhöhung des Krankheitsgrads. Dies gilt insbesondere nach einem Sturzereignis und der einschneidenden Konsequenz der hüftnahen Fraktur [4, 5]. Die Erforschung der Alltagsgehfähigkeit dieser Zielgruppe ist nun von einer Evidenzlücke geprägt, die auf methodischen und forschungsethischen Herausforderungen basiert [9]. Hier bietet sensorbasiertes Bewegungsmonitoring (SB) eine individuelle Beobachtungsmöglichkeit direkt am Körper [6], die sich für die Probanden ohne gesundheitliche Folgen, Einschränkungen oder besondere Anstrengungen gestaltet und auch in deren Lebenswirklichkeit zum Einsatz kommen kann [9]. Durch SB können fehlende Werte im Mobilitätsassessment reduziert werden [6]. Es bietet eine individualisierte Datenlage [13], die das Bewegungsverhalten veränderungssensitiv abbildet [20]. Diese Art der Beobachtung ist auch bei kognitiven Einschränkungen, wie demenziellen Erkrankungen, möglich [13]. Auch handelsübliche Fitnesstracker, beispielsweise der Marke Garmin, werden in der medizinischen und gesundheitswissenschaftlichen Forschung verwendet [18]. Diese eignen sich zur Erfassung der körperlichen Aktivität über die Variable Schritte pro Tag (SpT) [11]. Der Tragekomfort aktueller Geräte ermöglicht es, den Fitnesstracker unterbrechungsarm und ohne Einschränkungen für den/die Patient/-in am Handgelenk zu platzieren [1]. In der Fachliteratur besteht die Forderung, dass die Messung von Rehabilitationsleistungen auch für Patient/-innen nachvollziehbar und relevant sein sollte [6]. Die Kennzahl SpT ist eine Größe, die nicht nur von den Patient/-innen, sondern auch von den Pflegenden sowie Angehörigen, die eine wichtige Rolle in der Förderung körperlicher Aktivität Rolle spielen [22], verstanden werden dürfte. Auf dieser Basis könnten sich nachvollziehbare Handlungsanweisungen zur Mobilisierung bzw. Mobilität ableiten lassen [2]. In verschiedenen Einsatzszenarien (Laufband, Teststrecke, 24-h-Verlauf) wies der Sensor des Garmin vívofit® 3 (Garmin Ltd. oder deren Tochtergesellschaften, Schaffhausen, Schweiz) die höchste Übereinstimmung mit einem Forschungspedometer auf (*r* = 0,90) [3]. Im Hinblick auf das Gehen mit und ohne Hilfsmittel besteht die Vermutung, dass die SpT in der geriatrischen Zielgruppe aufgrund des Gangbilds mit < 10 % der Schrittzahl eher unterschätzt, aber nicht überschätzt werden [14, 25]. Die Sensortechnologie wird in der vorliegenden explorativen Studie genutzt, um Erkenntnisse über die Mobilität, in Form von SpT, der Zielgruppe im Rehabilitationsprozess nach einer hüftnahen Fraktur zu gewinnen. Die Forschungsfrage der Studie lautet: Wie verhält sich die Schritteentwicklung nach der Operation einer hüftnahen Fraktur auf der geriatrischen Traumastation und darüber hinaus?

## Methodik

### Design

Die explorative Beobachtungsstudie wurde im Längsschnitt von 10 Wochen an geriatrischen Traumapatient/-innen nach der operativen Versorgung einer hüftnahen Fraktur durchgeführt. Zu beobachtendes Verhalten waren SpT im Zeitverlauf, die durch das technische Hilfsmittel eines Fitnesstrackers (vívofit® 3 von Garmin) registriert und gespeichert wurden.

### Stichprobe

Es konnten 20 Patient/-innen (Frauenanteil 80 %, Durchschnittsalter 85,2 Jahre ± 7,86) aufgenommen werden. Das Statistische Bundesamt berichtete 2018 von 182.080 Patient/-innen mit den ICD-10‑4 Diagnosen S72.0- bis S72.9 im Alter von 65 bis > 95 Jahren [24]. In das Alterstraumaregister der Deutschen Gesellschaft für Unfallchirurgie (AltersTraumaRegister DGU®) wurden 2018 6873 Fälle aufgenommen. Die Auswahlgesamtheit der hier beschriebenen Studie beläuft sich auf 87 Patient/-innen, die im Rekrutierungszeitraum (Juni 2018 bis Februar 2019) auf einer nach den Kriterien der DGU zertifizierten geriatrischen Traumastation behandelt wurden [10].

### Instrumente

Der genutzte Fitnesstracker beinhaltet einen 3‑Achsen-Beschleunigungssensor, der bei einem Armbandumfang von 13,5 bis 20,5 cm und einem Gewicht von 26 g am Handgelenk getragen wird. Er sammelt Informationen, die in die Berechnungen eines Algorithmus einfließen, der die Art der Bewegung abschätzt und auf diese Weise Schritte zählt. Die Bewegung wird klassifiziert, indem Signalmerkmale extrahiert werden. Dies sind z. B. Zeitintervalle, Frequenzen oder statistische Merkmale, welche durch Kombination eine möglichst exakte Aufzeichnung für die intendierte Zielbestimmung ermöglichen [7]. Die aufgezeichneten Schritte wurden zunächst im Fitnesstracker selbst gespeichert; zur Auswertung wurden die Daten dann auf einen Computer geladen. Die dabei exportierte .csv-Datei enthielt Datenpunkte über die Zeit und die absolute Schritteanzahl. Die Batterielaufzeit des genutzten Trackers beläuft sich auf mindestens ein Jahr; Daten werden bis zu 4 Wochen gespeichert [15, 16]. Die Wahl der Gerätschaft orientierte sich an den Anforderungen des Studiendesigns: Längsschnitterhebung im häuslichen Umfeld der Patient/-innen. Kriterien [18] wie Wasserdichtigkeit, Robustheit, Aussehen und Tragekomfort, vergleichbar mit einer Armbanduhr [1], waren bei der Auswahl von besonderer Bedeutung, um Bedingungen zu schaffen, die zu einer möglichst langen und durchgehenden Studienteilnahme motivieren und Belastungen vermeiden. Weitere Faktoren betrafen Sensorart, Messgenauigkeit, Batterieleistung sowie Preis und Zugänglichkeit. Voreinstellungen, wie beispielsweise Vibrationsalarme, wurden ausgestellt, um technisch programmierte Stör- oder Motivationsfaktoren, die eine Bewegungsaufforderung darstellen, zu vermeiden.

### Durchführung

Im Rekrutierungszeitraum wurden alle Patient/-innen oder deren rechtliche Vertreter nach der Operation einer hüftnahen Fraktur über die Studie informiert. Die Rekrutierung ohne Ausschlusskriterien sollte eine Datenbasis schaffen, in die Werte verschiedener Mobilitätslevel einfließen. Das Anlegen des Trackers erfolgte sodann unter Einbezug der Informationen über das Geburtsjahr und Geschlecht. Der Datenschutz wurde durch Pseudonymisierung und Anonymisierung sichergestellt. Es erfolgten regelmäßige Besuche auf der Station sowie in der Rehabilitationseinrichtung und im häuslichen Umfeld, um die Daten auszulesen.

### Analyse

Die deskriptive Datenanalyse beinhaltet die Verteilung der Variablen SpT, Zeit in Form von Tagen sowie die individuellen wöchentlichen Schrittedurchschnitte von einer Woche (M7) bis 10 Wochen (M70) nach Beginn der Beobachtung (t0). Wenn an einzelnen Beobachtungstagen fehlende Werte vorlagen, floss die Wochen-Schrittezahl der betreffenden Person nicht in die Analyse ein, um beim Bilden des Durchschnittswerts mögliche tagesabhängige Ausreißer zu beachten. t0 und der letzte Tag der Beobachtung (tn) wurden nicht in die Analyse einbezogen, da das An- und Ablegen des Trackers nicht immer zur gleichen Tageszeit erfolgen konnten. Der individuelle Zeitabstand zwischen dem Tag der Hospitalisierung (TdH) und t0 wurde herangezogen, um den durchschnittlichen Beobachtungsbeginn zu ermitteln. Davon ausgehend konnte im Zeitverlauf von 10 Wochen (M7–M70) die entsprechende tatsächlich vergangene Zeit dargestellt werden.

## Ergebnisse

Der Beobachtungsbeginn liegt durchschnittlich bei 8,8 Tagen (±3,76, *Med* = 8, *n* = 20) nach TdH. Somit sind zu den Werten von M7 bis M70 rund 9 Tage hinzuzurechnen, um die tatsächlich verstrichene Zeit nach TdH, zu der die Schrittewerte (Tab. 1) auftreten, abschätzen zu können. Die Variable M7 bildet den Zeitraum Tag 1 bis Tag 7 der Beobachtung ab. Dies entspricht für die Patient/-innen in etwa Woche 1 bis 2 nach TdH. M70 entspricht den Wochen 10 und 11 nach TdH. Die deskriptiven Statistiken der SpT für die Zeiträume sind in Tab. 1 aufgeführt. Im Gesamtmittel erfolgt von M7 bis M70 ein Anstieg um den Faktor 1,285 (±0,351). Der Mittelwert liegt zu M7 bei 353,57 SpT (±310,15) und steigt zu M70 auf 2482,07 (±1374,12). Die höchsten Anstiege um das je 1,8-Fache zeigen sich von M14 (*M* = 556,27 ± 478,11) auf M21 (*M* = 1024,86 ± 921,24) sowie von M42 (*M* = 1268,21 ± 880,47) auf M49 (*M* = 2367,14 ± 1680,08). Auch von M7 (*M* = 353,57 ± 310,15) auf M14 (*M* = 556,27 ± 478,11) liegt ein Anstieg um das 1,573-Fache vor. Von M35 (*M* = 1199,37 ± 1078,73) auf M42 sowie M49 auf M56 (*M* = 2387,95 ± 1236,65) stagnieren die Mittelwerte der SpT. Geringfügige Abstiege zeigen sich von M28 (*M* = 1208,27 ± 1210,45) auf M35 (0,99-Fach) und von M63 (*M* = 2689,98 ±2339,71) auf M70 (0,92-Fach). Die Spannweite der SpT gestaltet sich im Zeitverlauf der Beobachtung sehr breit. Maxima übersteigen den Median durchschnittlich um das 3,4-Fache (±1). Der Median liegt durchschnittlich beim 7,2-Fachen (±4,5) des Minimums. Im Beobachtungsverlauf zeigt sich eine Verringerung dieser Unterschiede, wobei parallel dazu die Fallzahl sinkt.

**Table 1:** 

Zeitvariable	TdB	Woche nach TdH	SpT					
			MW	SD	Median	Min	Max	*n*
M7	1–7	1–2	353,57	310,15	313,07	61,43	1311,29	16
M14	8–14	2–3	556,27	478,11	485,71	119,43	1769,43	12
M21	15–21	3–4	1024,86	921,24	532,43	38,00	2632,43	13
M28	22–28	4–5	1208,27	1210,45	836,21	93,29	3926,29	12
M35	29–35	5–6	1199,37	1078,73	940,43	55,86	3501,86	12
M42	36–42	6–7	1268,21	880,47	1159,71	285,29	3214,43	11
M49	43–49	7–8	2367,14	1680,08	2245,07	540,57	6189,14	10
M56	50–56	8–9	2387,95	1236,65	2406,50	913,43	4690,43	8
M63	57–63	9–10	2689,98	2339,71	2286,93	365,14	7664,43	8
M70	64–70	10–11	2482,07	1374,12	2428,79	445,00	4230,71	8

*MW* Mittelwert, *SD* „standard deviation“ (Standardabweichung), *Min* Minimum, *Max* Maximum, *n* gültige Fälle, für die an 7 Beobachtungstagen Daten vorliegen, *TdB* Tag der Beobachtung, *TdH* Tag der Hospitalisierung, *SpT* Schritte pro Tag

## Diskussion

Im Gesamtmittel kommt es zu einer Verdopplung der Schritteanzahl zwischen M7 (≙1 bis 2 Wochen nach TdH) und M21 (≙3 bis 4 Wochen nach TdH). Zwischen M28 und M42 (≙4 bis 7 Wochen nach TdH) kommt es zu einer Stagnation, bevor es nach rund 7 Wochen zu einem erneuten Anstieg um das 1,8-Fache kommt, worauf die Schritteentwicklung bis zum Ende der Beobachtung erneut eher stagniert. Es sind stets hohe Spannweiten sowie Standardabweichungen zu verzeichnen. Die Maxima unterscheiden sich im Beobachtungsverlauf im Durchschnitt um mehr als das Dreifache vom Median. Die Hälfte der Patient/-innen geht in Anbetracht der Minima im Beobachtungsverlauf durchschnittlich über 7‑mal mehr Schritte. Das lässt auf das Vorhandensein mehrerer Gruppen der Schritteentwicklung schließen, die es zukünftig zu explorieren gilt. Allerdings ist auch die sinkende Fallzahl zu beachten. Es muss in Betracht gezogen werden, dass Patient/-innen mit größeren gesundheitlichen und Einschränkungen häufiger fehlende Werte aufweisen könnten oder eher aus der Studie ausscheiden.

Inwieweit die vorliegenden Werte plausibel für Patient/-innen nach Hüftfrakturen sind, kann anhand dreier Studien [8, 12, 21] eingeordnet werden. Während der Zeit auf der Station liegen die SpT bei Davenporth et al. [8] (*n* = 20, Frauenanteil 90 %, Durchschnittsalter 79,1 ± 9,3 Jahre) niedriger (*M* = 35,7 SpT ± 80,4, *M*in = 0, *Ma*x = 626, *N* = 20) als in der vorliegenden Studie bei 1 bis 2 Wochen nach TdH (*M* = 353,6 SpT ± 310,2, *Min* = 61,4, *Max* = 1311,3). Davenporth et al. haben ihre Untersuchung auf einer orthopädischen Station durchgeführt. Dagegen begann die Beobachtung der vorliegenden Studie auf einer speziellen geriatrischen Traumastation. Es gibt einen signifikanten Vorteil im Behandlungserfolg der Mobilisierung auf der geriatrischen Traumastation [26], der in der Regel über Assessmentwerte überprüft wird. Zukünftig könnte dies auch durch einen Vergleich von SpT abgebildet werden. Die Werte der Stichprobe von Davenporth et al. rangieren zwischen 0 und 626 SpT; die Standardabweichung ist sehr hoch [8]. Dies spricht dafür, dass einzelne Patient/-innen dort zu einer Gruppe gehören könnten, die eine gute Erholung bzw. Mobilisierung zeigen, obwohl sie nur auf einer orthopädischen Station behandelt werden. Fleig et al. [12] (*n* = 49, Frauenanteil 63,3 %, Durchschnittsalter 79,5 ± 7,8 Jahre) machen in einem nicht näher bestimmten Zeitraum von maximal 12 Monaten nach der Fraktur einen Median von 2467,7 SpT aus, der für einen Beobachtungszeitraum ≥ 3 Tagen (8 h/Tag) gilt. Ausschluss erfolgte bei demenziellen Erkrankungen oder Heimbewohnern sowie bei schlechter Gehfähigkeit. Ab 7 bis 8 Wochen nach TdH liegen die SpT in der vorliegenden explorativen Studie innerhalb dieser Werte. Eine genauere Zeiteinordnung der Studie von Fleig et al. würde erlauben abzuschätzen, inwieweit die Entwicklung der täglichen Schritte bereits nach 2 Monaten abgeschlossen wäre. Des Weiteren wäre zu überprüfen, inwiefern dies für verschiedene Subgruppen gelten würde. O’Halloran et al. [21] führten ihre Interventionsstudie in einem Rehabilitationsprogramm durch (*n* = 30, Frauenanteil 84 %, Durchschnittsalter 82,6 ± 5,2 Jahre). Ausschluss erfolgte bei kognitiven Beeinträchtigungen oder einem schlechten Allgemeinzustand sowie hohen Aktivitätsniveaus. Vor und nach einer 8‑wöchigen Intervention, in der eine Gruppe ein spezielles motivierendes Interview neben der Standardbehandlung erhielt, wurden die SpT beobachtet. Zu Beginn liegen sie in der Interventionsgruppe (IG) bei durchschnittlich 4279 SpT (±2915) und in der Kontrollgruppe (KG) bei durchschnittlich 4093 SpT (±2712). Das sind Werte, die in der vorliegenden Stichprobe von einzelnen ab 7 bis 8 Wochen nach TdH erreicht werden. Auch das lässt vermuten, dass die Entwicklung bereits ab 2 Monaten stagnieren könnte. Betrachtet man die Werte bei O’Halloran et al. nach der Intervention, so fällt auf, dass die IG durchschnittlich 4788 SpT (±3158) aufweist und sich in der KG durchschnittlich 3388 SpT (±2379) beobachten lassen. Ohne die besondere Motivation in der IG tritt in der KG eine signifikante Verschlechterung der Ausgangslage ein. Dieser Befund ergänzt die Annahme, dass es 8 Wochen nach Hospitalisierung zu einem Stagnieren der Schritteentwicklung kommen könnte, um die Frage, ob es für eine oder mehrere Subgruppen von Patient/-innen sogar wieder zu einem Absinken der SpT kommen könnte. Wenn Patient/-innen nach einer erfolgreichen stationären geriatrischen Mobilisierung und folgenden rehabilitativen Weiterbehandlung wieder ins häusliche Umfeld zurückkehren, könnte sich ein eher immobilisierendes Verhalten einstellen.

Der Median der SpT gesunder alternder Menschen (*Med* = 69 Jahre) liegt bei 7496,07 SpT (IQR 4493,58 [106,0–20828,3]) [19]. Auf Grundlage eines systematischen Reviews englischsprachiger Studien, die sich mit Schritten beschäftigten, wird für gesunde Erwachsene über 65 Jahren eine tägliche Schritteanzahl zwischen 5000 und 6000 Schritten empfohlen und für Erwachsene mit chronischen Erkrankungen oder körperlichen Einschränkungen zwischen 1500 und 4500 SpT [27]. Die Verteilung der SpT in der vorliegenden Studie lässt vermuten, dass einzelne Patient/-innen diese Werte innerhalb der ersten 2 Monate nach TdH erreichen, ein Großteil jedoch nie.

Der Vergleich der Schrittzahlen aus der vorliegenden Studie mit Ergebnissen ähnlicher Untersuchungen zeigt, dass die beobachtete Anzahl der Schritte plausibel ist. Für Personen, die sich am besten erholen, darf angenommen werden, dass sich mit fortschreitender Rehabilitationszeit ihre körperliche Aktivität nicht nur in SpT, sondern auch durch andere Aktivitäten, wie etwa Radfahren, ausgedrückt werden könnte. Somit besteht die Möglichkeit eines Unterschätzens der tatsächlichen Bewegung aufgrund der Wahl der Kennzahl. Aktivitäten, die über Gehen, Laufen oder Ruhen hinausgehen, können nicht eindeutig erfasst werden [17]. Auch der Einsatz von Hilfsmitteln kann zu einer Unterschätzung der SpT führen [14]. Am 7. Tag postoperativ sind 75 % der Patient/-innen (*n* = 6804) aus dem ATR gehfähig [23]. Ein Großteil dieser Gruppe kann gestützt (10 %) oder mit Hilfsmitteln wie Rollatoren (26 %), Gehbock (14 %) oder Gehwagen (24 %) gehen. Nur 1 % kann selbstständig gehen. 22 % haben noch keine Gehfähigkeit erreicht.

Insgesamt darf, begründet durch das Gangbild, angenommen werden, dass bei geriatrischen Patient/-innen durch den Algorithmus des Geräts weniger Schritte gespeichert werden als tatsächlich getätigt wurden. Der Fehler liegt gemäß einer experimentellen Studie mit einer hinsichtlich der Alters- und Geschlechtsstruktur vergleichbaren Stichprobe, jedoch ohne Fraktur, bei < 10 % der SpT. Dies trifft für Proband/-innen mit und ohne Einschränkungen der Gehfähigkeit zu. Beim Gehen mit Stock oder Rollator kann das Unterschätzen jedoch höher ausfallen [14]. Es wäre also möglich, dass die SpT sowohl bei hohem als auch bei geringem Rehabilitationserfolg unterschätzt wurden. Der Fehler ließe sich durch das Tragen des Sensors an der Ferse oder an der Hüfte zwar verringern [14], jedoch hat das Tragen des Motion-Trackers am Handgelenk wie eine Armbanduhr den Vorteil, dass dieser leicht und ohne Hilfe anzulegen ist und somit vollständig in den Alltag integriert werden kann. Wie sich der Fehler im häuslichen Umfeld verhält, insbesondere unter Einbezug von Hilfsmitteln und Komorbiditäten, muss in zukünftigen Studien untersucht werden [25]. In Abgleich mit den SpT aus den oben beschriebenen Studien [8, 12, 21] scheinen die Werte der vorliegenden explorativen Studie jedoch plausibel. Die vorliegenden Daten können durch clusteranalytische Verfahren unter Einbezug der Werte des geriatrischen Assessments sowie der Wohnsituation und dem Einsatz von Hilfsmitteln weitere Erkenntnisse über Entwicklungsverläufe nach hüftnahen Frakturen liefern.

### Limitationen

Um die Aussagekraft der vorliegenden Untersuchungsergebnisse zu erhöhen, wären u. a. folgende Änderungen hinsichtlich des Studiendesigns notwendig:

- Angleichung des Beobachtungsbeginns,
- Vermeidung eines frühzeitigen Abbruchs,
- Einbezug von Daten über die Rehabilitationseinrichtung (Beginn, Ausscheiden, Dauer, Art),
- Einbezug von Daten zum Hilfsmitteleinsatz (z. B. Rollatoren),
- regelmäßige Messung der Schrittlänge und Anpassung in der Applikation,
- Einbezug von Informationen über die Trageseite und dominante Körperhälfte.

## Ausblick

Sensorbasiertes Bewegungsmonitoring (SB) eignet sich zur Schaffung von Grundlagen für individualisierte Interventionen. Die explorative Analyse der SpT könnte weiterhin durch clusteranalytische Verfahren ergänzt werden, um klar abgrenzbare Entwicklungsverläufe darstellen zu können. Hiermit könnten Patient/-innen hinsichtlich ihrer SpT in Gruppen eingeteilt werden, um deren Charakteristiken weiterzuuntersuchen. Aufgrund der hohen Bedeutung von Bewegung und Mobilität in der geriatrischen Behandlung sollte SB auch für weitere Morbiditäten explorativ eingesetzt werden, um eine Datengrundlage für SpT zu generieren.

## Fazit für die Praxis

- Sensorbasiertes Bewegungsmonitoring ist für die geriatrische Zielgruppe geeignet.
- Fitnesstracker-Armbänder eignen sich für den Einsatz bei der geriatrischen Zielgruppe, auch im häuslichen Umfeld.
- Mobilisierung und Immobilität können durch Schritte pro Tag ausgedrückt werden.

## References

[1] Altenbuchner A, Haug S, Kretschmer R, Schreier G, Hayn D (2018). How to measure physical motion and the impact of individualized feedback in the field of rehabilitation of geriatric trauma patients. Health informatics meets ehealth: biomedical meets eHealth—from sensors to decisions. Proceedings of the 12th eHealth Conference.

[2] Altenbuchner A, Haug S, Weber K, Hayn D, Eggerth A, Schreier G (2019). Exploratory analysis of motion tracking data in the rehabilitation process of geriatric trauma patients. dHealth 2019—from eHealth to dHealth. Proceedings of the 13th Health Informatics Meets Digital Health Conference.

[3] An H-S, Jones GC, Kang S-K (2017). How valid are wearable physical activity trackers for measuring steps?. Eur J Sport Sci.

[4] Barth A, Doblhammer G, Mayer T (2017). Physische Mobilität und Gesundheit im Alter. Die transformative Macht der Demografie.

[5] Becker S, Becker S, Brandenburg H (2014). Gerontologie – eine interdisziplinäre Wissenschaft. Lehrbuch Gerontologie. Gerontologisches Fachwissen für Pflege- und Sozialberufe; eine interdisziplinäre Aufgabe.

[6] Benzinger P, Lindemann U, Becker C (2014). Geriatric rehabilitation after hip fracture. Role of body-fixed sensor measurements of physical activity. Z Gerontol Geriat.

[7] Bieber G (2014). Methodik zur mobilen Erfassung körperlicher Aktivität mittels Beschleunigungssensoren.

[8] Davenport SJ, Arnold M, Hua C (2015). Physical activity levels during acute inpatient admission after hip fracture are very low. Physiother Res Int.

[9] Deutsche Akademie der Wissenschaften (2015). Medizinische Versorgung im Alter. Welche Evidenz brauchen wir?.

[10] Deutsche Gesellschaft für Unfallchirurgie, AltersTraumaZentrum DGU (2020). Kriterienkatalog. Version 1.3.

[11] Dominick GM, Winfree KN, Pohlig RT (2016). Physical activity assessment between consumer- and research-grade accelerometers: a comparative study in free-living conditions. JMIR Mhealth Uhealth.

[12] Fleig L, McAllister MM, Brasher P (2016). Sedentary behavior and physical activity patterns in older adults after hip fracture: a call to action. J Aging Phys Act.

[13] Fleiner T, Haussermann P, Mellone S (2016). Sensor-based assessment of mobility-related behavior in dementia: feasibility and relevance in a hospital context. Int Psychogeriatr.

[14] Floegel TA, Florez-Pregonero A, Hekler EB (2017). Validation of consumer-based hip and wrist activity monitors in older adults with varied ambulatory abilities. J Gerontol A Biol Sci Med Sci.

[15] GARMIN (2019) Technische Daten des vívofit 3. https://www8.garmin.com/manuals/webhelp/vivofit3/DE-DE/GUID-FA3C58BF-1124-4DE5-82CB-0DD6F461EF2E.html. Zugegriffen: 1. Nov. 2019

[16] GARMIN (2020) vívofit® 3. Technische Daten. https://buy.garmin.com/de-DE/DE/p/539963#specs. Zugegriffen: 30. Mai 2020

[17] Garriguet D, Tremblay S, Colley RC (2015). Comparison of Physical Activity Adult Questionnaire results with accelerometer data, Canada.

[18] Henriksen A, Haugen Mikalsen M, Woldaregay AZ (2018). Using fitness trackers and smartwatches to measure physical activity in research: analysis of consumer wrist-worn wearables. J Med Internet Res.

[19] Kiselev J, Nuritdinow T, Spira D (2019). Long-term gait measurements in daily life: results from the Berlin Aging Study II (BASE-II). PLoS ONE.

[20] Nicolai SE (2011). Sensorbasierte Messung und Bedeutung Sensorbasierte Messung und Bedeutung körperlicher Aktivität bei Patienten nach Hüftfraktur in der geriatrischen Rehabilitation.

[21] O’Halloran PD, Shields N, Blackstock F (2016). Motivational interviewing increases physical activity and self-efficacy in people living in the community after hip fracture: a randomized controlled trial. Clin Rehabil.

[22] Sauter A, Curbach J, Rueter J (2019). German senior citizens’ capabilities for physical activity: a qualitative study. Health Promot Int.

[23] Sektion Alterstraumatologie DGU (2019). Jahresbericht 2019 – AltersTraumaRegister DGU® für den Zeitraum bis Ende 2018.

[24] Statistisches Bundesamt (2018) Gesundheit. Tiefgegliederte Diagnosedaten der Krankenhauspatientinnen und -patienten 2017. https://www.destatis.de/DE/Themen/Gesellschaft-Umwelt/Gesundheit/Krankenhaeuser/Publikationen/Downloads-Krankenhaeuser/tiefgegliederte-diagnosedaten-5231301177015.html. Zugegriffen: 16. Okt. 2019

[25] Straiton N, Alharbi M, Bauman A (2018). The validity and reliability of consumer-grade activity trackers in older, community-dwelling adults: a systematic review. Maturitas.

[26] Taraldsen K, Thingstad P, Sletvold O (2015). The long-term effect of being treated in a geriatric ward compared to an orthopaedic ward on six measures of free-living physical behavior 4 and 12 months after a hip fracture—a randomised controlled trial. BMC Geriatr.

[27] Tudor-Locke C, Craig CL, Brown WJ (2011). How many steps/day are enough? For adults. Int J Behav Nutr Phys Act.

